# Cross-Talk between Oxidative Stress and Inflammation in Preeclampsia

**DOI:** 10.1155/2019/8238727

**Published:** 2019-11-04

**Authors:** Marilene Brandão Tenório, Raphaela Costa Ferreira, Fabiana Andréa Moura, Nassib Bezerra Bueno, Alane Cabral Menezes de Oliveira, Marília Oliveira Fonseca Goulart

**Affiliations:** ^1^Faculdade de Nutrição, Universidade Federal de Alagoas, Campus A.C. Simões, BR 104 Norte, Km 96, 7, Tabuleiro dos Martins, CEP 57.072-970 Maceió, Alagoas, Brazil; ^2^Instituto de Química e Biotecnologia (IQB/UFAL), Rede Nordeste de Biotecnologia (RENORBIO), Universidade Federal de Alagoas, Campus A.C. Simões, BR 104 Norte, Km 96, 7, Tabuleiro dos Martins, CEP 57.072-970 Maceió, Alagoas, Brazil; ^3^Programa de Pós-graduação em Ciências da Saúde, ICBS, Universidade Federal de Alagoas, Campus A.C. Simões, BR 104 Norte, Km 96, 7, Tabuleiro dos Martins, CEP 57.072-970 Maceió, Alagoas, Brazil

## Abstract

The occurrence of hypertensive syndromes during pregnancy leads to high rates of maternal-fetal morbidity and mortality. Amongst them, preeclampsia (PE) is one of the most common. This review aims to describe the relationship between oxidative stress and inflammation in PE, aiming to reinforce its importance in the context of the disease and to discuss perspectives on clinical and nutritional treatment, in this line of research. Despite the still incomplete understanding of the pathophysiology of PE, it is well accepted that there are placental changes in pregnancy, associated with an imbalance between the production of reactive oxygen species and the antioxidant defence system, characterizing the placental oxidative stress that leads to an increase in the production of proinflammatory cytokines. Hence, a generalized inflammatory process occurs, besides the presence of progressive vascular endothelial damage, leading to the dysfunction of the placenta. There is no *consensus* in the literature on the best strategies for prevention and treatment of the disease, especially for the control of oxidative stress and inflammation. In view of the above, it is evident the important connection between oxidative stress and inflammatory process in the pathogenesis of PE, being that this disease is capable of causing serious implications on both maternal and fetal health. Reports on the use of anti-inflammatory and antioxidant compounds are analysed and still considered controversial. As such, the field is open for new basic and clinical research, aiming the development of innovative therapeutic approaches to prevent and to treat PE.

## 1. Introduction

Preeclampsia (PE) is one of the most common gestational complications, being clinically characterized by a systolic blood pressure of 140 mmHg or higher, a diastolic blood pressure of 90 mmHg or higher, or both systolic and diastolic blood pressure above ≥140/90 mmHg, measured twice with a four-hour interval, with proteinuria in 24 h urine ≥ 300 mg or protein/creatinine ratio ≥ 0.3. In the absence of quantitative methods, a 1+ test tape in the urine proteins may be used [1–3]. However, in the absence of proteinuria, PE is diagnosed associated with elevated blood pressure levels, using the following criteria: thrombocytopenia (number of thrombocytosis less than 100,000/*μ*L), renal insufficiency (serum creatinine above 97 *μ*mol/L), reduced liver function (enzymatic activity AST and ALT two times higher than the reference range limit, being for AST ≥ 60 UI/L and for ALT ≥ 64 UI/L), and pulmonary edema, cerebral, or visual disturbances [4].

Although the pathophysiological mechanisms of PE remain obscure, it is known that placental changes occur early in pregnancy, associated with an imbalance between the generation of reactive oxygen species (ROS) and the antioxidant defence system, characterizing oxidative stress. There is also a generalized inflammatory process, as well as the presence of progressive vascular endothelial damage, which culminates in placental dysfunction [5]. Despite this, it is not well established if the oxidative stress is the result of generalized oxidative cellular damage, which can affect proteins, lipid membranes, and deoxyribonucleic acid (DNA), caused by the disease already established, or if it precedes the clinical establishment of PE, being involved in its pathogenesis [6, 7].

Considering the above, this review aims to describe the relationship between oxidative stress and inflammation in PE, contributing to reinforce their importance in the context of the disease and to discuss perspectives on clinical and nutritional treatment in this research line.

## 2. Pathophysiology of PE

### 2.1. Trophoblastic and Placental Invasion: Normal Gestation *vs.* PE Gestation

The placenta, a highly complex membranous vascular organ, is developed during gestation and is responsible for the metabolic interaction between the mother and fetus, such as transport of oxygen and nutrients, fetal metabolite elimination and the production of hormones, as human chorionic gonadotropin hormone, estrogen, progesterone, and human placental lactogen [8, 9]. It has a diameter of 15 to 17 cm and an approximate weight of 500 g in a term gestation. Its growth is proportional to the gestational period; i.e., the placenta grows as the pregnancy progresses [10]. Some pathological conditions can trigger placental insufficiency, such as hypertension, diabetic vasculopathy, and anatomical disorders. Thus, changes in maternal homeostasis may modify placental structure and function and thereby affect fetal growth [10].

In the development of healthy gestation, the trophoblastic cells are assigned to invade the maternal endometrium and cause remodeling of the spiral arteries, aiming to increase their calibers and consequent supply of oxygenated blood and nutrients to the placenta [11, 12]. In women who develop PE, an abnormal trophoblastic invasion occurs early in pregnancy, which implies poor oxygenation of the intervillous space and persistence of the primary characteristics of the uterine spiral arteries, maintaining their high resistance. Thus, because there is no remodeling of these arteries, there is less oxygenated blood supply and nutrients, causing the placenta to be reduced in size. This process of hypoxia/reperfusion is always marked by an exacerbated production of ROS, when oxygen molecules are reintroduced into the tissue, after occurrence of hypoxia, leading to oxidative stress [13, 14].

Among the mechanisms proposed to explain the relationship between hypoxia and the ROS presence, problems during the aerobic cellular respiration are initially included. Thus, as oxygen (O_2_) concentration decreases within the cell, there is also a decrease in oxidative phosphorylation and lower formation of adenosine triphosphate (ATP), which result in dysfunction on several intracellular systems [15]. Besides, an increase in the production of ROS occurs through mitochondria, which can lead to lesion and/or cell death [16, 17].

In addition, a possible rise in cytosolic calcium, caused probably by the ischemia/reperfusion process, leads to the activation of the protease calpain, responsible for promoting the breakdown of a peptide bridge of the enzyme xanthine dehydrogenase, leading to the formation of the enzyme xanthine oxidase, which in turn, requires oxygen to perform the transformation of hypoxanthine into xanthine. In the ischemia stage, therefore, accumulation of these two substances occurs. With reperfusion, the hypoxanthine is oxidized to xanthine—which is increased in women with PE [16, 18, 19]—and subsequently in uric acid, generating as byproducts of this reaction the superoxide radical anion (O_2_^•−^)—which may be generated by electron capture in the mitochondrial transport system or via cyclooxygenase in the metabolism of arachidonic acid [20, 21]—hydrogen peroxide, and, in the presence of divalent ions, such as iron and copper, hydroxyl radicals (^•^OH) [16]. In this way, the activation of nonspecific proteases and phospholipases occurs, in response to the rise in intracellular calcium, during reperfusion, and results in the synthesis of proinflammatory mediators, such as platelet activating factor, leukotrienes, thromboxanes, and prostaglandins [22].

Thus, the presence of both trophoblast and placenta, and not necessarily the fetus, is essential for the PE development, since the disease also affects molar pregnancies (where genetically abnormal placental tissue proliferates in the absence of the fetus, giving rise to tumors and gestational trophoblastic disorders). In addition, the greater the placental mass, as in multiple pregnancies, the greater the risk of developing this disease, because the placenta is covered by a syncytiotrophoblast, a type of multinucleated cell with high invasive potential that will aid in the embryo implantation in the maternal endometrium. When there is some type of damage in these cells, trophoblastic fragments are released into the maternal circulation, with signaling to the endothelial cells, which phagocyte them in normal situations. However, in PE, there is also a compromise in the activity of endothelial cells, which makes it difficult to remove these fragments, and although it is not clear which placental factor is responsible for triggering PE, it is known that the increase of trophoblastic fragments in the circulation is related to the onset of the disease [23].

Regarding the changes in placental architecture, considering the morphological and functional characteristics, placentas from pregnant women with PE present diameter, thickness, weight, volume, cord size, and number of cotyledons smaller than usual. In addition, areas with bruise, infarction, and clot presence, with a marginal and paracentral cord insertion, in comparison with normal placentas, which present insertion of the central cord, are present [24–26].

### 2.2. PE *vs.* Oxidative Stress

Normal pregnancy is characterized as a prooxidant period, where ROS production occurs, characterizing oxidative stress, with reduced plasma levels of free antioxidants and increased purine catabolism. In many pregnancy-related disorders, including PE, this prooxidant characteristic is even more exacerbated [27].

Thus, scientific evidence suggests that reduced perfusion due to impaired trophoblastic invasion and aberrant placentation triggers a condition of oxidative stress in the placenta by the following mechanisms that increases O_2_^•−^ formation: (a) perfusion that can lead to repeated hypoxia/reoxygenation, a potent stimulus for the activation of the xanthine oxidase and the nicotinamide adenine dinucleotide phosphate oxidase (NADPH oxidase) [28–30]; (b) stimulation of the electron transport chain by the hypoxia/reperfusion and electron transport chain by the hypoxia/reperfusion, specifically complexes I and III [31]. Upon addition, in the mitochondrial matrix, of manganese superoxide dismutase (MnSOD) or copper and zinc superoxide dismutase (CuZnSOD) in the intermembrane space, the conversion of O_2_^•−^ to hydrogen peroxide (H_2_O_2_) is catalyzed, followed by its reduction to water by glutathione peroxidase (GPx) or catalase (CAT) [32, 33].

**Figure 1:**
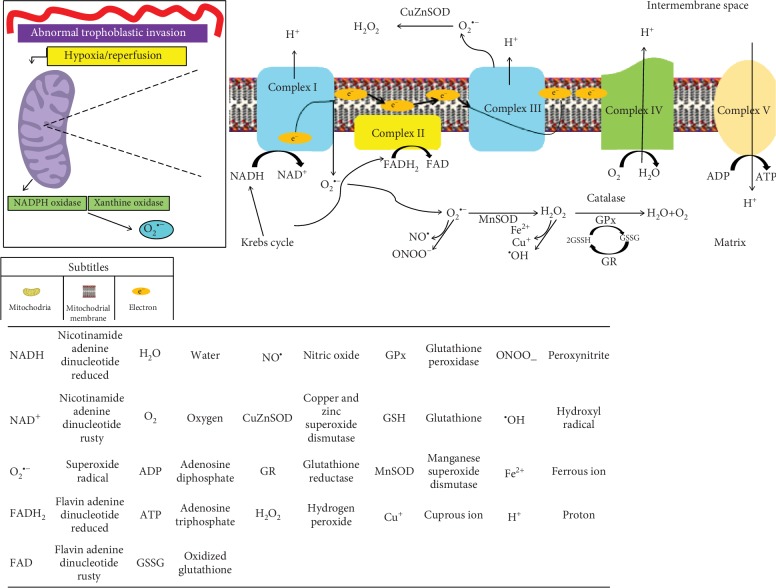
Mitochondrial production of some reactive oxygen species. The reduced perfusion due to impaired trophoblastic invasion triggers a condition of oxidative stress in the placenta by some mechanisms: (a) perfusion that can lead to repeated hypoxia/reoxygenation, a potent stimulus for the activation of the xanthine oxidase and nicotinamide adenine dinucleotide phosphate oxidase (NADPH oxidase), enzymes that are important precursors in the formation of O_2_^•−^ [28–30]; (b) the hypoxia/reperfusion also stimulates the electron transport chain, specifically complexes I and III [31], which increases the O_2_^•−^ production [28]. In the mitochondrial matrix, manganese superoxide dismutase (MnSOD) or copper and zinc superoxide dismutase (CuZnSOD) in the intermembrane space catalyzes the conversion of O_2_^•−^ to hydrogen peroxide (H_2_O_2_). H_2_O_2_ can then be completely reduced to water by antioxidant enzymes, such as glutathione peroxidase (GPx) or catalase (CAT) [32, 33]. Adapted from Yiyenoğlu et al. [28], Redman [29], Poston et al. [30], Chamy et al. [32], and Raijmakers et al. [33].

Figure 1 illustrates this process in the mitochondria.

The increase of oxidative stress in PE may also occur due to the increase in the circulating levels of tumor necrosis factor alpha (TNF-*α*), which, indirectly, may be able to regulate, in a positive way, lectin-like oxidized low-density lipoprotein (LDL) receptor-1 (LOX-1). This regulation results in increased uptake of oxidized LDL, leading to increased O_2_^•−^ production, via activation of NADPH oxidase. Several compounds present in the plasma of women with PE can also activate LOX-1, such as phospholipids, platelets, cytokines, and apoptotic cell fragments, resulting in increased oxidative stress, confirmed by increased expression of O_2_^•−^ and peroxynitrite (ONOO^−^); this last one is able to upregulate the LOX-1 expression, suggesting the presence of a feedback mechanism, in which LOX-1 activation induces oxidative stress, which in turn induces LOX-1 [34, 35].

### 2.3. PE *vs.* Inflammation and Endothelial Dysfunction

With the increase of oxidative stress in PE, a concomitant increase of the inflammatory response occurs, through the cytokines' production, such as TNF-*α* and interleukin- (IL-) 6, which led to a reduction in the anti-inflammatory cytokine production, such as IL-10, with consequent cell damage [14, 36]. Additionally, the process of hypoxia/reperfusion culminates with greater production of ROS, reactive nitrogen species (RNS), and lipid peroxides, while the antioxidant defence is reduced, including superoxide dismutase (SOD), glutathione peroxidase (GPx), and catalase (CAT), leading to an increased systemic oxidative stress condition, also including damaged DNA, low-density lipoprotein (LDL) oxidation, and reduction in melatonin production [37].

**Figure 2:**
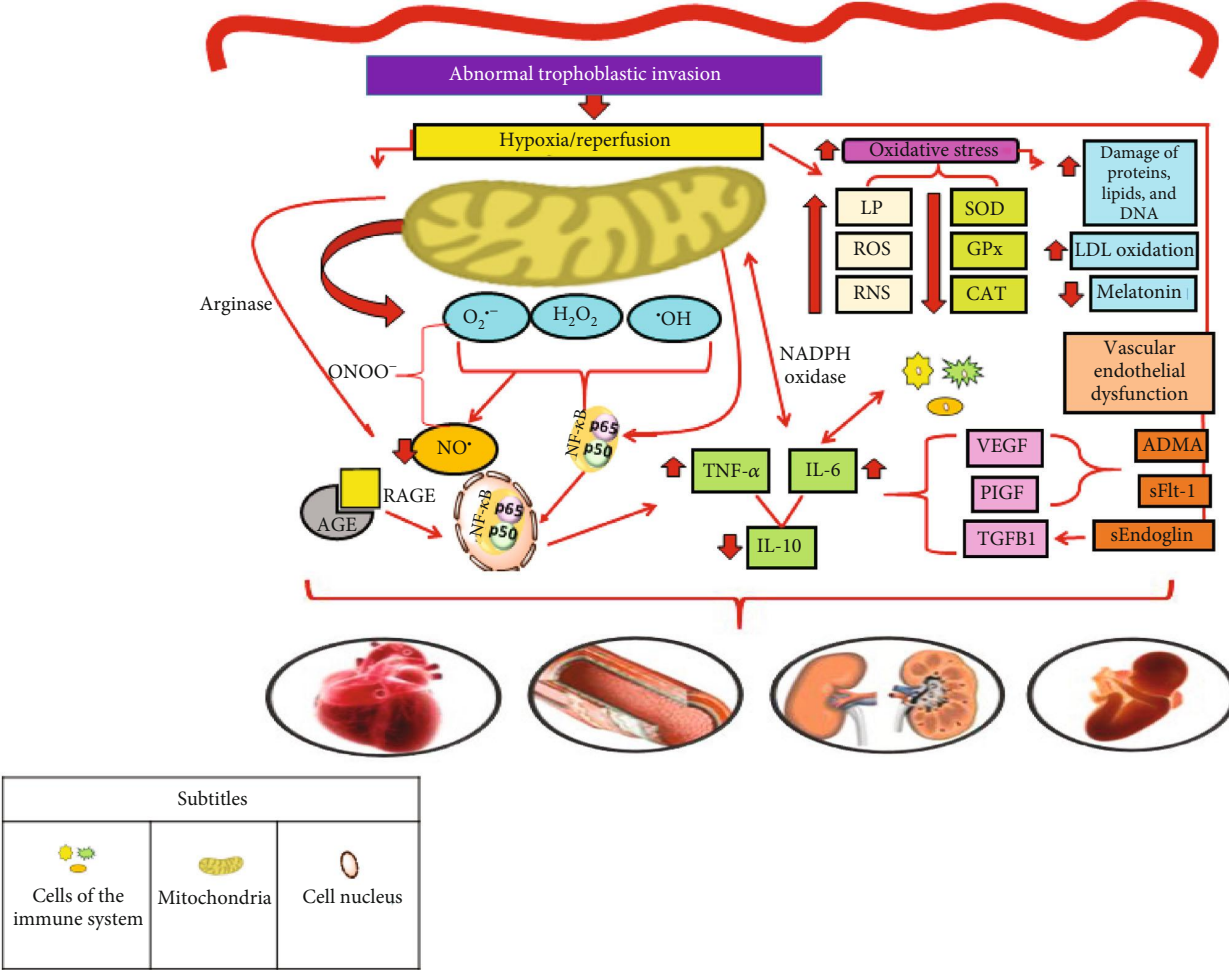
Mechanisms suggested in the pathophysiology of preeclampsia. The process of abnormal trophoblastic invasion, which culminates in repeated episodes of hypoxia/reperfusion, leads to the oxidative stress in PE. In turn, the process of hypoxia/reperfusion culminates with greater production of reactive oxygen species (ROS), reactive nitrogen species (RNS), and lipid peroxides, while the antioxidant defence is reduced, including SOD, GPx, and CAT, leading to an increased systemic oxidative stress condition, besides other factors resulting from oxidative stress, including damaged DNA, low-density lipoprotein (LDL) oxidation, and reduction in melatonin production [37]. Thus, there is a concomitant increase of the inflammatory response, through the cytokine production, such as tumor necrosis factor alpha (TNF-*α*) and interleukin- (IL-) 6, which led to a reduction in the anti-inflammatory cytokine production, such as IL-10, and, consequently, cell damage [14, 36]. In the inflammatory response, there is the involvement of genes related to oxidative stress, especially the nuclear factor kappa B (NF-*κ*B), located in the cellular cytoplasm. ROS are able to oxidize the I*κ*B kinase (IKK) complex, leading to the release of NF-*κ*B, which is formed by p50 and p65 subunits. Because it is a nuclear factor, the NF-*κ*B molecule enters the cell nucleus and promotes the transcription of several proinflammatory cytokines such as IL-6 and TNF-*α*. This process occurs naturally during gestation, but in the PE, its action is exacerbated [39, 96]. Advanced glycation end products (AGEs), resulting from the glycation of proteins or other biomolecules, interact with their receptors (RAGEs) located in a wide variety of tissues. Such interaction is responsible for triggering the activation of several signaling pathways, culminating with the activation of NF-*κ*B, leading to an inflammatory process [52, 53]. Beyond the inflammatory process, PE also results in endothelial dysfunction due to reduced bioavailability of nitric oxide (NO^•^) and increased production of placental antiangiogenic factors, such as dimethylarginine (ADMA), sEndoglin (soluble endoglin), and Fms-like receptor tyrosine kinase (sFlt-1). The association of these changes leads to health consequences, such as cardiovascular-, endothelial-, renal-, and fetal-related complications [38]. Legend: ADMA: dimethylarginine; AGE: advanced glycation end products; CAT: catalase; GPx: glutathione peroxidase; IL: interleukin; LP: lipid peroxides; NADPH oxidase: nicotinamide adenine dinucleotide phosphate oxidase; NF-*κ*B: nuclear factor kappa B; ONOO^−^: peroxynitrite; PIGF: placental growth factor; RAGE: advanced glycation end product receptors; RNS: reactive nitrogen species; ROS: reactive oxygen species; sFlt-1: soluble Fms-like receptor tyrosine kinase; SOD: superoxide dismutase; TGFB1: transforming growth factor beta; TNF-*α*: tumor necrosis factor alpha; VEGF: vascular endothelial growth. Adapted from Sanchéz-Araguren et al. [14], Harmon et al. [36], Chiarello et al. [37], Cheng et al. [38], Striz et al. [39], Rayman et al. [96], Sargent et al. [52], and Guedes-Martins et al. [53].

In the inflammatory response, there is involvement of genes related to oxidative stress, especially the nuclear factor kappa B (NF-*κ*B), located in the cellular cytoplasm. ROS are able to oxidize the I*κ*B kinase (IKK) complex, leading to the release of NF-*κ*B, which is formed by p50 and p65 subunits. As it is a nuclear factor, the NF-*κ*B molecule enters the cell nucleus and promotes the transcription of several proinflammatory mediators, such as the intracellular adhesion molecule 1 (ICAM-1) and the vascular cell adhesion molecule 1 (VCAM-1), along with proinflammatory cytokines such as IL-6 and TNF-*α*. This process occurs naturally during gestation, but in the PE, its action is exacerbated [38, 39] (Figure 2).

In addition to the aforementioned mechanism, during the trophoblastic invasion itself, the decidua, which is the lining of the uterus responsible for the formation of the maternal placenta portion, contains a large number of immune cells, such as macrophages, natural killer (NK) cells, T cells, and regulatory T cells (Treg), necessary to promote trophoblast migration. In PE, an immunological imbalance is observed, which results in the secretion of proinflammatory cytokines and decrease of Treg cells, this imbalance being responsible for the activation of a chronic inflammatory response in the immune system [36, 40].

During the PE, cells of the immune system (T-helper cells) are in high levels and secrete IL-17, which in turn stimulates TNF-*α* and IL-6, which induce the macrophage and neutrophil secretion. Macrophages, neutrophils, and proinflammatory T cells are also able to convert molecular oxygen into O_2_^•−^ by the phagocyte oxidase system, catalyzed by NADPH oxidase. Once activated, neutrophils can cause placental damage by the release of lysosomal enzymes and ROS. Thus, the activation of NADPH oxidase can be induced by lipoproteins and cytokines, such as proinflammatory interleukins and TNF-*α* [41].

**Figure 3:**
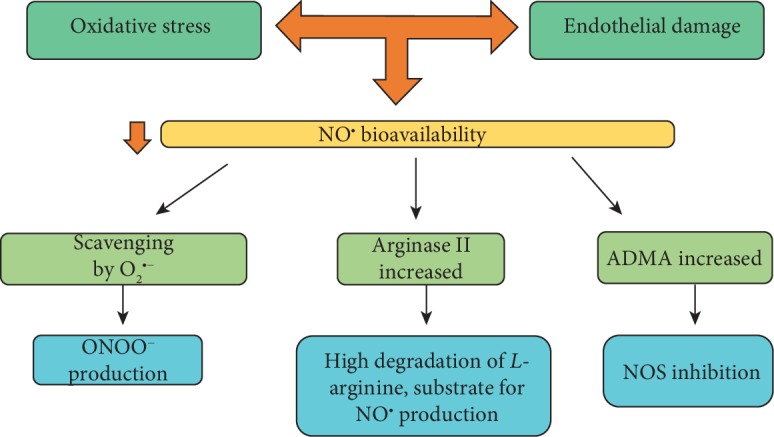
Routes through which the bioavailability of nitric oxide decreases in preeclampsia. Some pathways can contribute to the lower bioavailability of NO^•^ in the PE. The first one involves ROS, where it is suggested that O_2_^•−^ captures NO^•^ for the formation of peroxynitrite (ONOO^−^), which has a high redox potential [13]. In addition, ONOO^−^ reacts with lipids, leading to lipid peroxidation (LP) and generation of malondialdehyde (MDA) and its conjugates [97]. The second path involves the increase in the production of the enzyme arginase, responsible for catalyzing the conversion of *L*-arginine to *L*-ornithine and urea [98]. Therefore, the bioavailability of arginine for NO^•^ formation is compromised [99, 100]. The last one involves the presence of ADMA, an endogenous inhibitor of the enzyme nitric oxide synthase (eNOS), which is increased in PE and is able to decrease the synthesis of NO^•^ [14]. Legend: ADMA: dimethylarginine; NOS: nitric oxide synthase. Adapted from Sanchéz-Araguren et al. [14], Sankaralingam et al. [13], Takacs et al. [97], Rabelo et al. [98], Coman et al. [99], and Morris Jr. [100].

PE also results in endothelial dysfunction due to reduced bioavailability of nitric oxide (NO^•^) and increased production of placental antiangiogenic factors, such as dimethylarginine (ADMA), sEndoglin (soluble endoglin), and Fms-like receptor tyrosine kinase (sFlt-1), a soluble receptor formed by an alternative splicing, leading to the loss of the transmembrane portion of Flt-1, a common receptor for angiogenic factors [42]. NO^•^ is a vasodilator agent capable of promoting smooth muscle relaxation, regulating endothelial function, platelet aggregation, and the development of muscle cells [43].  Figure 3 shows three pathways capable of explaining the lower bioavailability of NO^•^ in the PE.

It is noteworthy that pilot studies, such as by Groten et al. [44], where, in a clinical trial, the NO^•^ donor drug penterythriltetranitrat (PETN) was supplemented to assess its prophylactic role in abnormal placentation. Significant improvement in uteroplacental perfusion was observed compared with the placebo (mean 1, 26 ± 0.36 vs. 1.49 ± 0.44; *p* < 0.01). In addition, a reduction in the frequency of preterm births, PE, and intrauterine growth restriction was observed, showing the beneficial action of this compound in preventing adverse outcomes of pregnancies in these cases.

Additionally, the endothelial dysfunction that exists in the PE is probably due to hypoxia/reperfusion, which causes oxidative stress that provokes placental production of a large number of antiangiogenic factors, such as sFlt-1 and sEndoglin, and reduction of angiogenic factors vascular endothelial growth factor (VEGF) and placental growth factor (PIGF). Thus, sFlt-1 binds to these circulating molecules and prevents these angiogenic factors from connecting to their common receptors on the cell membrane, causing dysfunction in vascular endothelial repair [45].

Additionally, the endothelial dysfunction also occurs due to an increase in endothelin-1 [46] expression and stimulation of the expression of autoantibodies to the angiotensin II type 1 receptor (AT1-AA). Differently from what occurs in a normal pregnancy, where there is a reduced sensitivity of the endothelium to angiotensin II (Ang II), in pregnant women with PE, due to genetic, immunological, and external factors, there is an excessive sensitivity to Ang II. Thus, AT1 receptor stimulation is also elevated in disease. In addition, women with PE produce autoantibodies to the AT1 receptor (AT1-AA), and the scientific literature suggests that the increase of such antibodies leads to hypertension, from complement activation, proteinuria, and increased levels of antiangiogenic factors [47].

In this context, Lei et al. [48] evaluated the association between AT1-AA and hypertension using meta-analysis, as well as the prognosis of AT1-AA for hypertensive diseases, and confirmed that elevated levels of AT1-AA in the serum of women are significantly associated with hypertensive disorder, especially PE. In turn, Szpera-Gozdziewicz et al. [49] investigated AT1-AA levels in pregnant women with chronic hypertension, gestational hypertension, and PE compared with healthy pregnant women, showing that women with gestational hypertension and PE presented higher levels than the others.

It is noteworthy that AT1-AA are detectable in animal models of PE and are responsible for elevation of sFlt-1 and soluble endoglin, oxidative stress, and endothelin-1, all of which are enhanced in preeclamptic women [47]. Inhibition of AT1-AA, using the inhibitory peptide n7AAc, prevents the increase in maternal blood pressure and several pathophysiological factors associated with PE in rats, being recognised as a potential therapy for PE [50].

Considering the occurrence of hypoxia/reperfusion in the pathophysiology of PE, which leads to increased oxidative stress, as well as the role of oxidative stress in triggering the inflammatory process observed in the disease, the stimulus that AT1-AA exerts on smooth vascular muscle cells with the consequent activation of NF-*κ*B generates a vicious cycle between oxidative stress and inflammation [47, 51].

Advanced glycation end products (AGEs), resulting from the glycation of proteins or other biomolecules, interact with their receptors (RAGEs) located in a wide variety of tissues. Such interaction is responsible for triggering the activation of several signaling pathways, culminating with the activation of NF-*κ*B, leading to an inflammatory process. In the PE, considering the ongoing inflammatory process, the possible increase in the AGE/RAGE expression culminates with a higher production of ROS, through the activity of NADPH oxidase, increased stimulation of NF-*κ*B, with consequent release of O_2_^•−^ and its effect on NO^•^, resulting in the formation ONOO^−^, which worsens, even more, the existing oxidative stress. Although the data are insufficient to affirm, it is suggested that in PE, RAGEs may be increased, thus bringing more complications to the diseased women [52, 53].

Table 1 summarizes the main inflammatory factors involved in the pathophysiology of PE, as well as their forms of action.

## 3. PE *vs.* Oxidative Stress Biomarkers

Some oxidative stress biomarkers are commonly evaluated in studies involving pregnant women with PE, including antioxidant enzymes (CAT, SOD, and GPx), antioxidant compounds (reduced glutathione (GSH) and vitamins C and E), and products derived from the activity of ROS, mainly through lipid peroxidation (LP) (such as malondialdehyde, MDA) [54] and oxidation of DNA and proteins [55].

Table 2 presents the main oxidative stress markers, enzymes, and antioxidants, involved in the pathophysiology of this disease.

An extensively evaluated oxidative damage is LP, which plays an important role in the pathophysiology of PE. Oxidized lipids (LP products such as TBARS and F2-isoprostane) affect the functionality of antioxidant enzymes (GPx and SOD) as well as nonenzymatic antioxidants (vitamins A, C, and E) causing oxidative damage [56].

The relationship of PE and its outcomes with some oxidative stress biomarkers and antioxidant enzymes is summarized in Table 3. The results have shown that women with PE, compared to controls, present higher levels of oxidative stress and lipid peroxidation biomarkers, besides lower levels of antioxidant compounds.

It is worth mentioning that a relevant review on oxidative stress biomarkers was published in 2017 [57], aimed at discussing the detection of these markers (MDA, CAT, SOD, GPx, NO^•^, TBARS, and vitamins C and E) in biological fluids and at highlighting the need for further studies to validate their use in the prediction or diagnosis of pregnancy-related diseases, including PE. The authors concluded that the oxidative stress markers are promising for the identification of some complications of gestation; in addition, each one alone may have limitations, but when associated, they may help in the diagnosis of adverse conditions in pregnancy [57].

## 4. PE *vs.* Inflammatory Biomarkers

Several studies have reported higher levels of proinflammatory cytokines in the serum and plasma of PE women, compared with those with normal pregnancies [36, 58–60]. Although the literature provides wide and comprehensive reports related to this subject, the present review does not pretend to be exhaustive, considering that the key point of this study is to emphasize the interaction between the factors suggested as being involved in the pathophysiology of PE, and not only the inflammatory process [61] (Table 4). We are reporting only selected inflammatory markers, as there are several other cytokines and chemokines that have been studied in PE. The cytokines more widely studied and with a well-established knowledge of their mechanisms, in relation to PE, were chosen.

Table 4 shows that several studies have been conducted in humans and animals, especially in recent years, in order to clarify the role of inflammatory cytokines in PE. The inclusion or exclusion criteria were added. From these findings, it is clear their involvement in the pathophysiology of the disease and that their levels increase according to the severity of PE. In addition, experimental studies [40, 62, 63] also show an increase in levels of inflammatory factors in PE, which is associated with endothelial dysfunction, also observed, as well as the protective role of IL-10, when administered in an appropriate dose/time [64, 65].

## 5. PE *vs.* Antioxidant Therapy

The use of supplementation with antioxidant compounds in several clinical and pathological contexts has been widely discussed and performed; however, in the PE, their uses present controversial results both in prevention and in treatment [66].

Table 5 lists the majority of the reported studies on humans, involving oral antioxidant supplementation for the prevention and treatment of PE. The inclusion or exclusion criteria were added. The included studies were carried out with different antioxidant compounds, being the first reported in 2003. It is possible to observe that there is no consensus about the dosages and times of administration of the supplementation, corroborating with controversial findings in relation to the outcomes of pregnancy. However, among the natural antioxidants tested in PE, lycopene appears to play a crucial role in neonatal outcomes, including increased mean birth weight, reduced rates of intrauterine growth restriction (IUGR), and PE [67, 68].

Other antioxidant compounds such as selenium [69], allicin [70], coenzyme Q10 [71], *N*-acetylcysteine [72], *L*-arginine [73, 74], and vitamins C and E [75] have also been tested for the prevention and/or treatment of PE. However, the results are controversial, with evidence of null influence [70, 72, 75] and beneficial action [69, 71, 73, 74] of antioxidants (Table 5). However, there is still insufficient evidence to recommend their use [76, 77]. In addition, a meta-analysis conducted by Tenório et al. [78] aimed to determine whether oral antioxidant therapies, of various types and doses, were able to prevent or treat women with preeclampsia. In this study, antioxidant therapy had no effects in the prevention of PE but did show beneficial effects in intrauterine growth restriction, when used in the treatment of this condition.

Additionally, *in vitro* studies have been developed with other antioxidant compounds, such as resveratrol and melatonin, from cells involved in the pathogenesis of PE. The results were positive, suggesting their potential therapeutic use in the prevention and/or treatment of the disease [79–82]. The review of Kerleya et al. [83] discusses the use of ergothioneine as a possible mitochondrial target antioxidant, focusing on its physical properties, potential mechanisms of action, safety profile, and administration in relation to pregnancies complicated by PE.

Magnesium sulphate, widely used in clinical practice for the prevention of seizures in women with PE, has been studied in diseases involving the increase of oxidative stress by its potential action as an antioxidant, especially at the cellular and molecular levels, in addition to studies in animals and humans, with positive results. Thus, with further investigation, this compound may also be a preventive and/or therapeutic option for PE [84, 85].

In addition, other studies have evaluated the role of a mix of vitamins and minerals, including antioxidants, administered as enriched foods such as milk and bars, to prevent the onset of PE in women at high risk or with low concentration of these compounds. Wibowo et al. [86] and Vadillo-Ortega et al. [87] found positive results. That is, there was a decrease in the risk of PE in the supplemented women. However, it must be taken into account that such mix contained nutrients that do not play antioxidant roles, being difficult to judge the real effect of the antioxidant content.

Finally, despite the relationship mentioned between AGEs, inflammation, and oxidative stress in PE, there are few studies that used antioxidant and anti-inflammatory compounds in an associated way to prevent/minimize the adverse consequences to mother and fetus health. Stupakova et al. [88], in turn, aimed to evaluate the inhibition of platelet aggregation and the possibility of correction with resveratrol and nicorandil, in a rat model with PE induced by L-NAME. The findings were positive in relation to the drugs tested in order to aid in the homeostasis of the affected animals.

## 6. PE *vs* Therapy with Anti-Inflammatory Compounds

The oral use of anti-inflammatory nutrients in the prevention and treatment of PE has been addressed by the scientific community [66] (Table 6), with emphasis on omega-3. However, systematic reviews have concluded that omega-3 supplementation in PE pregnancy does not present beneficial effects either in the prevention or in the control of the pressure levels in the disease [89, 90]. It is worth mentioning that PE has its origin in the initial period of pregnancy, even during the placentation process, and therefore, its prevention must be performed even before pregnancy has been established, which could justify the scarce results found in the literature [8, 15].

Currently, aspirin has been increasingly used to prevent PE. Although it consists of an inhibitor of platelet aggregation and a vasodilator, it also exerts an important anti-inflammatory action, acting in the reduction of prostaglandins and eicosanoids, thus reducing the inflammatory response [12, 91]. A meta-analysis performed by Askie et al. [92] evaluated the use of antiplatelet agents in the primary prevention of PE and observed an association of these with a moderate but consistent reduction in the relative risk of PE, as well as the occurrence of adverse effects.

Some studies are being conducted on aspirin and PE, many of which are reported on *ClinicalTrials.gov*. However, the results are still scarce and conflicting [93–95].

## 7. Conclusions

The supplementation with antioxidants, anti-inflammatory compounds, and nutrients has been considered in order to minimize the damage caused by oxidative stress and inflammation present in PE's pathophysiology. The results are still controversial. There is still no consensus on the best strategies for prevention and treatment of the disease, especially for the treatment of oxidative stress and inflammation, which are characteristics of the disease.

In view of the above, it is possible to establish an important relationship between oxidative stress and inflammatory process in the PE pathogenesis, considering that they are interconnected, acting on the various mechanisms involved in the disease. On the other hand, despite their relationship, the clinical and nutritional treatments described in the literature have not presented, so far, an effect, since they do not act on the cause of the disease, but in the sense of mitigating its consequences, not enough to prevent its progression. Thus, further research is urgently needed to elucidate the pathophysiology of this disease, in order to help health professionals, from the development of innovative therapeutic approaches to prevent and to treat PE, and to contribute to reduce the serious health effects of the mother-fetus binomial.

## Figures and Tables

**Table 1:** Inflammatory factors involved in the pathophysiology of preeclampsia.

Cytokines and transcription factors	Summary of the mechanisms of action
IL-1	Produced by macrophages and monocytes, as well as nonimmune cells, such as fibroblasts and activated endothelial cells, during cell injury, infection, invasion, and inflammation. There are two known types: IL-1*α* and IL-1*β*. They act on the same receptors, IL-1RI—considered the active receptor—and IL-1RII, which is functionally inactive. IL-1*α* is markedly associated with cell membranes and acts through cellular contacts. IL-1*β* is synthesized as a precursor protein (pro-IL-1*β*), which is not secreted in the active form, until it is metabolized by the caspase-1 enzyme. IL-1*β* produces systemic inflammation through the activation of COX-2, with the formation of PGE2 in the anterior hypothalamus. It also produces SP, NO^•^ (activating the enzyme nitric oxide synthase), and endothelial adhesion molecules, such as ICAM-1 and VCAM-1 [101, 102].

IL-2	Produced mainly by TCD4 cells and to a lesser extent by TCD8^+^ cells. It acts through IL-2R*α*, IL-2R*β*, and IL-2R*γ* receptors, using the intracellular JAK/STAT pathway to stimulate the growth and proliferation of T-lymphocytes and B-cells. It also induces the production of other cytokines, such as, for example, IFN-*γ* and lymphotoxin-*α* which results in the activation of monocytes, neutrophils, and natural killer cells [102, 103].

IL-6	Produced by macrophages, monocytes, eosinophils, hepatocytes, and glia, with TNF-*α* and IL-1 being potent inducers. It promotes neutrophil maturation and activation, macrophage maturation, and differentiation/maintenance of T-lymphocytes and natural killer cells. In addition, it activates astrocytes and microglia and regulates neuropeptide expression after neuronal injury, contributing to its regeneration [101].

IL-8	IL-8 induces cytoskeletal reorganization, changes in intracellular Ca^2+^ levels, integrin activation, granular protein exocytosis, and respiratory burst [104].

TNF-*α*	It acts in three different ways: endocrine, autocrine, and paracrine. On the adipocyte, it plays a regulatory role in relation to the accumulation of body fat, through the increase of lipolysis and inhibition of lipogenesis, from the blockade of acetyl-CoA synthase action. It has a role in lipid biosynthesis, with a decrease in lipoprotein lipase expression and a reduction in the synthesis of the glucose transporter to the GLUT-4 membrane, decreasing the uptake of glucose by the cells mediated by the action of insulin. This reduction of peripheral sensitivity to insulin causes an increase in hepatic glycogenesis, characterizing a hyperinsulinemic state. In addition, it has pleiotropic action; i.e., it is capable of influencing different cellular manifestations. It is still involved in the inflammation process, playing a major role in the cascade of cytokines and stimulating the synthesis of others [101, 105].

IL-17	This cytokine is predominantly produced by TCD4 lymphocytes with proinflammatory action, leading to the formation of IL-6 and IL-8 (chemokine) and the intercellular adhesion molecule in human fibroblasts. It is secreted from Th1 and Th17 cells, which are activated during immunological challenges, with cytotoxic potential to trigger inflammatory responses by recruiting immune cells, which release molecules of oxidative stress and, consequently, favor endothelial injury [36, 101].

NF-*κ*B	Signal-dependent transcription factor. For its activation, several second messenger systems may be required, thereby determining the induction of transcription of proinflammatory proteins. It can be activated by a variety of extracellular stimuli, such as proinflammatory cytokines (TNF-*α*, IL-1) [106].

Legend: Ca^2+^: calcium; COX-2: cyclooxygenase-2; GLUT: glucose transporter; ICAM-1: intercellular adhesion molecule 1; IL: interleukin; IFN: interferon; JAK/STAT: Janus family of tyrosinoquinases/transcription factors; NF-*κ*B: nuclear factor kappa B; NO^•^: nitric oxide; PE: preeclampsia; PGE: prostaglandin; SP: substance P; Th cells: T-helper cells; VCAM-1: vascular cell adhesion molecule 1.

**Table 2:** Principal biomarkers of oxidative stress and antioxidant compounds involved in the pathophysiology of preeclampsia.

Biomarkers of oxidative stress and antioxidant compounds	Definition
MDA	Derived from lipid peroxidation, through the breakdown of endocyclization of polyunsaturated fatty acids, containing more than two double bonds, such as linoleic, arachidonic, and docosahexaenoic acid [107].

CAT	It has at its active site the heme group and is attached to a peroxisome, an organelle responsible for cellular detoxification and oxidation of long chain fatty acids, an inexhaustible source of organic peroxides, carbonyl products, and singlet oxygen [107, 108].

SOD	The forms detected in humans are the Cu/ZnSOD located in the cytosol (dimeric), lysosomes, nucleus, and space between the inner and outer membranes of the mitochondria (tetramer), as well as the MnSOD located in the mitochondria [107, 109].

GPx	It is part of the selenoprotein group, with selenium, obtained through the diet linked to methionine, in foods of plant origin (selenomethionine) and cysteine bound in foods of animal origin (selenocysteine). This enzyme is capable of reducing peroxides to water or alcohol [107, 108].

NO^•^	It is a marker of reactive nitrogen species, being rapidly metabolized to stable products, i.e., nitrite and nitrate, in most body fluids, including plasma [110, 111].

TBARS	It is an indirect marker of lipid peroxidation; it measures the content of MDA [107, 110].

Vit. C	It acts as an antioxidant on ROS and RNS, in an aqueous biological environment, resulting in the formation of the radical anion semidehydroascorbate (Asc^•−^) or ascorbil, which is slightly reactive [107, 111].

Vit. E	The term vitamin E is the name of two different families of compounds: tocopherols and tocotrienols, capable of blocking the lipid peroxidation propagation step of polyunsaturated fatty acids from membranes and lipoproteins [111, 112].

Legend: CAT: catalase; Cu/ZnSOD: cooper and zinc superoxide dismutase; GPx: glutathione peroxidase; MDA: malondialdehyde; MnSOD: manganese superoxide dismutase; ROS: reactive oxygen species; RNS: reactive nitrogen species; SOD: superoxide dismutase; TBARS: lipid peroxidation products; vit. C: vitamin C; vit. E: vitamin E.

**Table 3:** Association studies of preeclampsia and its outcomes with levels of oxidative stress biomarkers and antioxidant enzymes, associated or not associated with the analysis of other biomarkers.

Study	Population	Inclusion/exclusion criteria	Biomarkers of oxidative stress/antioxidant enzymes	Outcomes
Atamer et al. (2005) [113]	NG (*n* = 25) CG (*n* = 28) PE (*n* = 32)	PE women had normal blood pressure during the first 20 weeks of gestation; no previous history of MD, RD, SAH, or DM; no history of antioxidant intake and medication of antihypertensive or aspirin; and no drugs at the time of collection of blood samples. Healthy women (control) did not suffer from medical conditions (DM or obesity), and none had a history of SGA or SAH, in any previous pregnancy.	MDA-serum MDA-placental SOD-erythrocyte CAT-erythrocyte GSH-Px-placental GSH-placental	Significant ↑ in serum and placental MDA levels in PE pregnant women, and a ↓ in GSH-Px and placental GSH levels.

Aydin et al. (2004) [114]	NT (*n* = 34) PE (*n* = 35)	Women not in labor, without ruptured membranes, neither multiple pregnancy nor medical complications, including autoimmune disorders, DM, inflammatory conditions, and no cases of SAH with superimposed PE. The NT were matched with those with PE for maternal age, gestational age at delivery, and gestational age at blood sampling.	MDA-plasma SOD-plasma NO-plasma	Significant ↑ in plasma MDA levels with increases in diastolic blood pressure (*p* < 0.001). In addition, SOD and NO levels were significantly ↓ with even greater reduction with ↑ diastolic blood pressure.

Yoneyama et al. (2002) [115]	NT (*n* = 26) PE (*n* = 26)	PE women had normal blood pressure, during the first 20 weeks of gestation; no previous history of CD, primary SAH, connective tissue disease, DM, or RD; no history of antioxidant intake and medication of antihypertensives or aspirin; well-established gestational age; no fetal structural anomaly; normal response to GTT; and no evidence of recent infection. NT matched for maternal age, parity, and gestational age.	MDA-plasma ADA-plasma	Significantly ↑ levels of MDA and ADA in PE (*p* < 0.05).

Orhan et al. (2003) [116]	NT (*n* = 16) PE (*n* = 9) GDM (*n* = 3)	Well-defined diagnosis of the diseases (PE and GDM) and healthy pregnant women in the NT group.	In plasma and erythrocytes TBARS GPx-Se GSH-Px CAT	GPx-Se activity significantly ↑ in pregnancy with insulin-dependent PE and GDM. In addition, simultaneous ↑ in plasma TBARS levels.

Hubel et al. (1996) [117]	PE (*n* = 8) NT (*n* = 9)	NT women without proteinuria or hyperuricemia. Exclusion criteria: patients with cigarette or illicit drug use, SAH, RD, or MD or previous history of lipid disorders.	Predelivery and postpartum: MDA-serum	The prepartum MDA levels were 50% ↑ in women with PE; ↓ in the postpartum period.

Serdar et al. (2003) [118]	Mild PE (*n* = 30) Severe PE (*n* = 30) NT (*n* = 50)	None of the patients had preexisting SAH or RD, hepatic, or hematologic diseases and were receiving any medication or vitamin supplementation until the study samples were taken. The NT had no signs of pregnancy complication, and all gave birth to healthy infants between 38 and 40 weeks of gestation.	In serum, placental and decidual Lipid peroxides Carbonylated proteins Vit. E Carotenoids	Lipid peroxides and carbonylated proteins significantly ↑ in the serum, placenta, and basal decidua, as well as vit. E and serum carotenoids were ↓ in severe PE women, compared to those with mild PE and NT. There was also a significant correlation between diastolic blood pressure and peroxylipids in the blood, placenta, and deciduous and serum carbonylated proteins.

Siddiqui et al. (2013) [119]	PE (*n* = 40) CG (*n* = 80)	Women were not in labor, without multiple pregnancies, and had neither ruptured membranes nor development of any simultaneous medical complications previously or during pregnancy, such as DM or inflammatory diseases. The CG did not exhibit any of the exclusion criteria. No vitamin supplements or aspirin was prescribed to the cases or controls in the month before their enrollment in the study.	MDA-serum Vit. E serum Total serum GSH	Markers of oxidative stress, including serum MDA, total GSH, and vit. E, and they were significantly ≠ in both groups, with ↑ levels of MDA, as well as ↓ levels of vit. E and total GSH, in women with PE.

Sahay et al. (2015) [120]	NT (*n* = 35) PE-PT (*n* = 11) PE-PPT (*n* = 14)	Inclusion criteria: individuals aged 18–35 years with a singleton pregnancy. Exclusion criteria: any other pregnancy complication (i.e., SAH, type 1 or type 2 DM, seizure disorder, RD, or LD), smoking, and drug or alcohol use. NT women aged 18–35 years, with a singleton pregnancy delivered at term and no medical or obstetric complications.	In placenta MDA GPx CAT	MDA levels were ↑ in all regions of the placenta between the PE and NT groups (*p* < 0.01). MDA levels were ↑ in the maternal central region relative to the fetal central region in the PE-PPT group (*p* = 0.023). MDA levels in the maternal central region were also ↑ in the preterm period than in the PE group (*p* = 0.014). CAT activity was ↓ in the maternal (*p* = 0.036) and fetal (*p* = 0.050) peripheral regions in the PE-PPT versus NT. The GPx activity was ↑ in the peripheral region than in the central fetal region in the NT group (*p* = 0.033).

Yuvaci et al. (2016) [121]	Severe PE (*n* = 32) Light PE (*n* = 30) CG (*n* = 37)	Inclusion criteria: age range of above 18 years and below 40 years and with a single live fetus in gestation at week 24 and above. CG had patients without any systemic disease. Exclusion criteria: history of PE in previous pregnancies, chronic disease, and drug use affecting renal and liver functions, SAH, GDM, type I or type II DM, connective tissue disease, RD and LD, hyper/hypothyroidism, hematologic disease, another reason for convulsions, a baby with abnormalities, multiple pregnancies, and who are found to have infection in the spot urinalysis.	Total thyroid-serum	Serum thiol levels were significantly ↓ in pregnant women with severe PE compared to those with mild PE and CG.

Lucca et al. (2016) [122]	Severe PE (*n* = 3) Light PE (*n* = 25) CG (*n* = 30)	Inclusion criteria: all pregnant women were in the 3^rd^ trimester of pregnancy and were in the same age group. Exclusion criteria: pregnant women with chronic diseases, infectious diseases, cancer, thyroid dysfunction, or any other disease, as well as smokers, drinkers, or anyone using any kind of medication, except for pregnant women, with PE who were using antihypertensives.	Thiol-plasma proteins and erythrocytes Vit. C-plasma CAT-plasma	Levels of TBARS present in plasma and erythrocytes were significantly ↑ in women with PE, whereas in thiolate protein groups, the amounts of vit. C and CAT were significantly ↓ in the female EP group when compared to those in the CG.

Legend: ADA: adenosine deaminase; CAT: catalase; CD: cardiovascular disease; CG: control group; DM: diabetes mellitus; GDM: gestational diabetes mellitus; GPx-Se: glutathione selenium peroxidase; GSH: glutathione; GSH-Px: glutathione peroxidase; GTT: glucose tolerance testing; LD: liver disease; MD: metabolic disorders; MDA: malondialdehyde; NG: not pregnant; NO: nitric oxide; NT: normotensive; PE: preeclampsia; PPT: preterm delivery; PT: birth at term; RD: renal disease; SAH: systemic arterial hypertension; SGA: small for gestational age; TBARS: lipid peroxidation products; vit. C: vitamin C; vit. E: vitamin E; ↑: higher; ↓: lower; ≠: different.

**Table 4:** Studies associating preeclampsia and its outcomes with levels of inflammatory markers in humans and animals.

Study	Population	Inclusion/exclusion criteria	Inflammation biomarkers	Outcomes
Cackovic et al. (2008) [58]	Humans: CG (*n* = 45) PE (*n* = 45)	Inclusion criteria: CG matched one to one for maternal and gestational age at enrollment and had a pregnancy course uncomplicated by PE. Exclusion criteria: preexisting proteinuria and/or SAH, active labor, clinical symptoms suggestive of viral or bacterial infection, known or suspected congenital malformation, and isolated IUGR.	Serum and urinary TNF-*α*	A ↑ serum concentration was observed in PE *vs.* CG as well as sFlt-1. On the other hand, urinary levels of TNF-*α* were ↓ in PE and did not correlate with the degree of proteinuria, when compared to CG. In addition, in PE, the fractionated excretion of TNF-*α* was significantly ↓ despite the existence of proteinuria. Thus, the ↓ renal excretion of TNF-*α* may contribute to the exacerbated inflammatory response, observed in the pathophysiology of the disease, considering that there is an accumulation of this biomarker in the body.

Sandrim et al. (2008) [59]	Humans: CG (*n* = 58) GDH (*n* = 56) PE (*n* = 45)	Inclusion criteria: women without preexisting SAH. Exclusion criteria: twin or multiple pregnancies or any evidence of previous medical illness.	Nitrite-serum	Serum levels of nitrite were ↓ in GDH and PE women *vs.* CG (-36% and -58%, respectively, both *p* < 0.05). Even ↓ serum concentrations of sEndoglin and sFlt-1 were observed in PE women *vs.* GDH and CG. Thus, impairment in the formation of NO^•^ in PE was suggested.

LaMarca et al. (2011) [62]	Experimental: Rats CG (*n* = 6) IG (*n* = 6)	—	IL-6	The results indicated a ↑ mean arterial pressure and AT1-AA in animals receiving chronic infusion of IL-6, which was abolished in those treated previously with the antihypertensive agent (losartan: angiotensin receptor antagonists). Thus, these data showed that IL-6 stimulates AT1-AA and that activation of AT1R mediates IL-6-induced hypertension during pregnancy.

Lai et al. (2011) [63]	Experimental: Rats CG (*n* = 6) IG (*n* = 6)	—	IL-10	Animals exposed to hypoxia tended to develop the characteristic symptoms of PE, such as placental injury, proteinuria, hypertension, and systemic symptoms. In addition, there was an ↑ in the expression of antiangiogenic factors, such as sFlt-1. However, after IL-10 administration, the protective role of this cytokine was observed, in relation to the development of symptoms and disease progression, indicating an IL-10 protective role in PE.

Dhillion et al. (2012) [40]	Experimental: Rats NP (*n* = 20) NP+IL-17 (*n* = 12) NP+tempol (*n* = 7) NP+IL-17+tempol (*n* = 11)	—	IL-17	IL-17 causes placental oxidative stress, which serves as stimulus modulating AT1-AAs that may play an important role in mediating IL-17-induced hypertension during pregnancy.

LaMarca et al. (2005) [64]	Experimental: Rats NP (*n* = 16) NP+ETa (*n* = 15) VR+TNF-*α* (*n* = 12) CG (*n* = 11)	—	TNF-*α*	Chronic infusion of TNF-*α* had no significant effect on arterial pressure or renal preproendothelin levels in virgin rats. These results suggest an important role for endothelin in mediating TNF-*α*-induced hypertension in pregnant rats.

Sahin et al. (2015) [123]	Humans: PE (*n* = 41) CG (*n* = 80)	Inclusion criteria: CG had all women in the 3^rd^ trimester of pregnancy and none of them developed PE or other pregnancy complications. Exclusion criteria: women in labour, multiple pregnancies, ruptured membranes, or medical history of SAH and DM.	IL-8	An enhanced inflammatory response was observed in severe PE women demonstrated by ↑ levels of IL-8 and decreased levels of IL-10. However, the intensity of platelet activation did not correlate directly with the change in plasma levels of IL-8 and IL-10 in PE women.

Silva et al. (2013) [65]	Humans: PE (*n* = 50) CG (*n* = 50)	Inclusion criteria: pregnant women in the 3^rd^ trimester with PE or with an uncomplicated pregnancy. Exclusion criteria: smokers and had systemic diseases and/or associated genital tract diseases (DM, SAH, RD, TD, LE, UI, and cervical or vaginal inflammation).	IL-6, IL-10, TNF-*α*, and IL-6/IL-10 ratio	The study reinforces the hypothesis that there is an immune dysfunction in PE, with an ↑ in the production of proinflammatory cytokines IL-6 and TNF-*α*, and a compensatory increase in IL-10.

Molvarec et al. (2015) [124]	Humans: PE (*n* = 59) CG (*n* = 60)	Inclusion criteria: all women were Caucasian and lived in the same geographic area. CG was in the early follicular phase of their menstrual cycle, and none of them received hormonal contraception. Exclusion criteria: multifetal gestation, SAH, DM, AID, angiopathy, RD, maternal or fetal infection, and fetal congenital anomaly. None of them was in active labor, and none had rupture of the amniotic membranes. Pregnant women with eclampsia or HELLP syndrome.	IL-17	The serum IL-17 levels are ↑ in PE, which may contribute to the development of the excessive systemic inflammatory response characteristic of the maternal syndrome of the disease.

Sun et al. (2016) [125]	Humans: PE (*n* = 160) CG (*n* = 140)	All women in the 3^rd^ trimester of pregnancy. Single pregnancy; cesarean section; same types of anesthesia; no previous medical history of SAH, CD, RD, DM, hyperthyroidism, or other complications that may lead to vascular disorders and hypoxic changes; and no infectious diseases.	IL-8	The IL-8 expression had positive association with the severity of PE. Results from enzyme-linked immunosorbent assay showed that the concentration of serum IL-8 in PE patients (180.27 ± 5.81 ng/L) was significantly higher than that in healthy controls (41.57 ± 5.67 ng/L). Patients with severe PE had even higher serum IL-8 levels.

Ribeiro et al. (2017) [126]	Humans: Early PE (*n* = 20) Late PE (*n* = 20) CG (*n* = 20)	Inclusion criteria: primiparous women without previous history of SAH or obstetric and medical complications. In CG, women with an uncomplicated pregnancy and matched for gestational age with the PE group. Exclusion criteria: multiple gestation, prior PE, illicit drug use, and medical conditions such as DM, cancer, SAH, acute infectious diseases, CD, AID, RD, and LD.	IL 4, IL-6, IL-17,IL-22, and TNF-*α*	Endogenous plasma levels of IL-6, IL-17, and TNF-*α* were significantly ↑ in the early-onset PE group than in the late-onset PE and normotensive groups, whereas IL-4 (Th2 profile) and IL-22 (Th17 profile) were not significantly different between the studied groups.

Aggarwal et al. (2019) [127]	Humans: PE+CG (*n* = 194) Mixed groups: I (*n* = 55) 28-36 weeks II (*n* = 139) 37 weeks onwards	PE diagnosis and CG not having any history of pregnancy-related complications, DM or any other chronic medical illness, vaginal bleeding throughout pregnancy, along with no evidence of congenital abnormalities, tuberculosis, and not having habits like tobacco, alcohol, and smoking.	TNF-*α*, IL-6, IL-4, and IL-10	The levels of TNF-*α* and IL-6 were significantly ↑ in PE cases, while the IL-4 and IL-10 were downregulated in comparison to control. In addition, a negative correlation was also observed between the two in PE (*p* = 0.0001).

Peixoto et al. (2016) [128]	Humans: CG (*n* = 30) PE (*n* = 16)	Inclusion criteria: women with PE, eclampsia, and HELLP syndrome, irrespective of gestational age and indication for delivery. The CG included pregnant women without complications, according to clinical and laboratory parameters. Exclusion criteria: pregnant women with gestational SAH, chronic SAH, PE superimposed on SAH, and patients who have had spontaneous premature delivery and PPRM.	IL-4, IL-10, IL-13, TNF-*α*, and IFN-*γ*	Patients with PE presented significantly ↓ placental levels of IL-10 and IL-13 than the CG. IL-4, TNF-*α*, and IFN-*γ* levels were similar on the two groups. ↑ inflammatory balance was observed in patients with PE *vs.* normal.

Kalantar et al. (2013) [129]	Humans: CG (*n* = 40) PE (*n* = 44)	Inclusion criteria: CG consisted of healthy women with uncomplicated pregnancy. They did not receive any special drug during the pregnancy except routine supplements. Exclusion criteria: pregnant women with pregestational DM and SAH.	TNF-*α*, IL-15, and IL-10	For PE women, significantly ↑ serum levels of TNF-*α* and IL-15, in comparison with CG. Conversely, the serum levels of IL-10 in CG were significantly ↑ *vs.* PE.

Kalinderis et al. (2011) [130]	Humans: CG (*n* = 30) PE (*n* = 30)	Inclusion criteria: PE developed during the 3^rd^ trimester of pregnancy. CG with no evidence of SAH or proteinuria during the current pregnancy and had no sign of gestational complication or fetal distress. Similar maternal age, gestational age and BMI to PE women. Exclusion criteria: women with multiple gestation, DM, SAH, infectious diseases in pregnancy, PPRM, active labour, polyhydramnios, and signs of other concurrent medical complications.	IL-1*β*, IL-6	Serum IL-6 and IL-1*β* levels were significantly ↑ in women with PE *vs.* CG.

El-Kabarity and Naquib (2011) [131]	Humans: CG (*n* = 60) PE (*n* = 120)	All pregnant women were in their 3^rd^ trimester, and CG were demographically matched to the PE women.	IL-12, IL-18	IL-18 was significantly ↑ in women with PE than in CG. IL-12 was not significantly ↑ in mild PE but significantly ↑ in severe cases.

Sharma et al. (2011) [132]	Humans: CG (*n* = 200) PE (*n* = 300)	Inclusion criteria: women in both the groups were primiparous, and the gestational age was between 25 and 36 weeks. All the selected women were nonsmokers and did not suffer from any active infectious process. Exclusion criteria: women associated with multiple pregnancies, DM, Rh-incompatibility, bleeding disorders, systemic LE, hydramnios, and pregnancies complicated by fetal abnormalities; pregnant women on any medication or chronic disorders, and metabolic disorders.	ET1, IL-2, TNF-*α*, and IFN-*γ*	↑ ET1, IL-2, TNF-*α*, and IFN-*γ* in the PE group *vs.* CG.

Legend: AID: autoimmune disease; AT1: autoantibody receptor against Ang II type 1; AT1-AA: angiotensin II receptor type 1; BMI: body mass index; CD: cardiovascular disease; CG: control group; DM: diabetes mellitus; ET1: endothelin-1; Eta: endothelin receptor antagonist; GDH: pregnant woman with hypertensive disorder; IFN: interferon; IG: intervention group; IL: interleukin; IUGR: intrauterine growth restriction; LD: liver disease; LE: lupus erythematosus; NP: normal pregnant; NT: normotensive; PE: preeclampsia; PPRM: preterm premature rupture of membranes; RD: renal disease; SAH: systemic arterial hypertension; tempol: 4-hydroxy-2,2,6,6-tetramethylpiperidine-*N*-oxyl; TD: thyroid disorders; TNF-*α*: tumor necrosis factor alpha; UI: urinary infection; VR: virgin rats; vit. E: vitamin E; sFlt-1: soluble Fms-like receptor tyrosine kinase; ↑: higher; ↓: lower.

**Table 5:** Human research involving oral antioxidant supplementation for the prevention and treatment of preeclampsia.

Study	Antioxidant	Inclusion/exclusion criteria	Dose/time	Outcomes
Banerjee et al. (2009) [67]	Lycopene	Inclusion criteria: primiparous women with singleton pregnancy between 12 and 20 weeks of gestation, with the absence of any medical problems, such as CH, RD, gross obesity, diabetes, thrombophilia, CD, or connective tissue disease. Criteria to PE: blood pressure consistently more than 140/90 mmHg in a previously normotensive woman accompanied with newly onset proteinuria of more than 300 mg/24 h urine collection or, >1+ on clean catch dipstick in a random urine sample in the absence of urinary infection.	2 mg 12-20 weeks until delivery	The supplemented group: ↑ in the incidence of adverse effects of preterm birth and LBW.

Pulido et al. (2016) [73]	*L*-Arginine	Inclusion criteria: women who had high-risk factors for developing PE (nulliparous, previous history of PE, CH, and BMI ≥ 30). Exclusion criteria: patients with multiple pregnancies or comorbidities (DM, hepatopathy, HD, and collagen disease), alcohol consumption, anti-inflammatory/antioxidant drug ingestion, infections, or concomitant medication.	3 g 3 weeks during gestation and 2 weeks postpartum	Maternal blood pressure and prematurity rates were significantly ↓ in the intervention group, while birth weight was ↑. The Apgar score < 7 to 5 min was not ≠ between the groups, and there was no neonatal or maternal death.

Valdivia-Silva et al. (2009) [74]	*L*-Arginine	Inclusion criteria: the patients were normotensive during the 1^st^ trimester and had no history of SAH. None of the women had a history of PE or other factors that cause IUGR. Exclusion criteria: women who had a history of being smokers, chronic diseases such as SAH, coronary HD, RD and/or DM, prophylactic treatments with aspirin or fetal malformations detected by ultrasonography, and the presence of some complication that would require an emergency delivery that has not allowed the treatment to continue for at least 3 weeks.	3 g 20 weeks until delivery	The risk of IUGR was 5x ↑ in infants born to mothers with PE without *L*-arginine therapy and twice as often in babies born to mothers with PE in the intervention group. The fetal biophysical profile and the Apgar score were significantly more favorable in the intervention group.

Teran et al. (2009) [71]	Coenzyme Q10	Inclusion criteria: women between 16 and 20 weeks of pregnancy (established by date of last menstrual period and confirmed by ultrasound), not currently taking medication, and with no known medical disorders. Exclusion criteria: women who were taking vitamin supplements.	200 mg 20 weeks until delivery	There were no ≠ between the groups in the incidence of LBW and mean birth weight. There was no perinatal mortality. Only 2 pregnancies resulted in preterm birth and both were in the placebo group.

Tara et al. (2010) [69]	Selenium	Inclusion criteria: gestational age up to 12 weeks, and with no indications for termination of the pregnancy. Exclusion criteria: in use of any drugs, except routine supplements of folic acid and ferrous sulphate, and a prior history or clinical features of any medical conditions, including thyroid disorders, DM, SAH, and infections.	100 *μ*g 1^st^ trimester until childbirth	Selenium supplementation significantly ↑ serum selenium concentrations in full-term newborns. However, there were no ≠ in systolic and diastolic blood pressure, total serum cholesterol and fractions, triglycerides, and high sensitivity C-reactive protein between the groups.

Aalami-Harandi et al. (2014) [70]	Allicin	Pregnant women at risk for PE, primiparous women, aged 18-40 years old who were carrying singleton pregnancy at 27 weeks of gestation.	400 mg garlic (1 mg allicin) 27 weeks, for 9 weeks	Significant protein ↓ in C-reactive protein and a significant ↑ in plasma GSH levels. No significant effect on serum lipid profiles, plasma levels of total antioxidant capacity and pregnancy outcomes.

Motawei et al. (2016) [72]	*N*-Acetyl-cysteine	Women with diagnosis of PE and health pregnancy to the control group.	400 mg For 6 weeks	Improvement in pregnancy outcomes, birth weight, and Apgar score among intervention group patients but without ≠ in the incidence of obstetric complications and markers of oxidative stress between the two groups.

Spinnato et al. (2007) [75]	Vits. C and E	Inclusion criteria: women who were 12 0/7 to 19 6/7 weeks pregnant and diagnosed with nonproteinuric chronic hypertension SAH or a prior history of PE in their most recent pregnancy that progressed beyond 20 weeks of gestation. Exclusion criteria: multifetal gestation, allergy to vit. C or E, requirement for aspirin or anticoagulant medication, 24-hour urinary protein 300 mg or more, prepregnancy DM, known fetal anomaly incompatible with life, or prior participation in the study.	1000 mg 400 IU 12-19 weeks until childbirth or disease development	Preterm membrane rupture was ↑ in the intervention group. There was no influence on the frequency of LBW, small for gestational age, stillbirth, birth measurement, asphyxia or Apgar scores.

Xu et al. (2010) [133]	Vits. C and E	Inclusion criteria: women between 12 and 18 completed weeks of pregnancy on the basis of last menstrual period and confirmed by early ultrasound examination. Exclusion criteria: women who regularly consumed supplements 200 mg/day for vit. C and/or 50 IU/day for vit. E; women who took warfarin; women who had known fetal abnormalities or known fetal chromosomal or major malformations in the current pregnancy; women who had a history of medical complications including endocrine disease, RD with altered renal function, epilepsy, any collagen vascular disease, active and chronic LD, HD, cancer, or hematologic disorder; women with repeated spontaneous abortion (women with a previous bleeding in the 1^st^ trimester were included if the site documented a viable fetus at the time of recruitment); and women who used an illicit drug during the current pregnancy.	1000 mg 400 IU 12-18 weeks until delivery	No difference in maternal adverse outcomes between groups, including rates of miscarriage, fetal death, neonatal death, preterm birth, IUGR, or small for gestational age.

Sharma et al. (2003) [68]	Lycopene	Primiparous women with gestation between 16 and 20 weeks with absence of any medical complication such as RD, SAH, HD, DM, or connective tissue disease.	2 mg 16-20 weeks until delivery or development of PE	The mean birth weight was ↑ and there was ↓ of IUGR in the intervention group.

Roberts et al. (2010) [134]	Vits. C and E	Inclusion criteria: pregnant women who had a singleton fetus with a gestational age of less than 16 weeks 0 days at the time of screening. Gestational age at randomization was between 9 weeks 0 days and 16 weeks 6 days. Women were eligible for inclusion if they had not had a previous pregnancy that lasted beyond 19 weeks 6 days. Gestational age was determined before randomization with the use of a previously described algorithm that took into account the date of the last menstrual period (if reliable information was available) and results of the earliest ultrasound examination. Exclusion criteria: women with elevated systolic blood pressure (135 mmHg or higher), elevated diastolic blood pressure (85 mmHg or higher), or proteinuria (300 mg of protein or more, as measured in a 24-hour urine sample, or a urine-dipstick result of 1+ or higher for protein), were taking or had taken antihypertensive medication, or were taking more than 150 mg of vitamin C or more than 75 IU of vit. E daily. DM that was present before the pregnancy, treatment with antiplatelet drugs or nonsteroidal anti-inflammatory agents, uterine bleeding within the week before recruitment, uterine malformation, serious medical condition, known fetal anomaly or aneuploidy, in vitro fertilization resulting in the current pregnancy, or abuse of illicit drugs or alcohol.	1000 mg 400 IU 9-16 weeks, until delivery	Vitamins C and E did not reduce adverse maternal or perinatal outcomes in women at high risk.

Legend: BMI: body mass index; DM: diabetes mellitus; GSH: reduced glutathione; HD: hearth disease; IUGR: intrauterine growth restriction; LBW: low birth weight; LD: liver disease; PE: preeclampsia; ↑: higher; ↓: lower; ≠: difference; RD: renal disease; SAH: systemic arterial hypertension; vits.: vitamins.

**Table 6:** Human research involving the oral supplementation of anti-inflammatory nutrients for the prevention and treatment of preeclampsia.

Reference	Anti-inflammatory	Inclusion/exclusion criteria	Doses and administration period	Outcomes
D'Almeida et al. (1992) [135]	Group 1: control Group 2: GLA, EPA, DHA Group 3: magnesium oxide	The patients were primiparous and multiparous and also had to be in the first four months of pregnancy to be eligible to enroll in the program.	7 mg 18 mg 10 mg 1000 mg In the first four months of gestation, for six months	↓: incidence of edema in group 2; group 3 had fewer individuals who developed hypertension during pregnancy. Three cases of eclampsia were reported in the control group.

Herrera et al. (1998) [136]	Intervention group: linoleic acid, calcium Control group: placebo (lactose)	Inclusion criteria: 1^st^ pregnancy, gestational age of 28–32 weeks, biopsychosocial risk score of 3 or more, positive roll-over test, and high MAP. Exclusion criteria: DBP of 80 mmHg or more at a previous prenatal visit, drug intake except for oral iron supplements, and history of SAH, CD, or RD.	450 mg 600 mg 600 mg 28-32 weeks until delivery	↓: incidence of PE in high-risk women *vs.* placebo group.

Bulstra-Ramakers et al. (1994) [76]	Intervention group: EPA and DHA Group control: placebo (coconut oil)	Inclusion criteria: birthweight below the 10th centile corrected for gestational age, parity, and sex in association with PIH. Birthweight below the 10th centile in association with RD. Birthweight below the 10th centile and placental abnormalities suggestive of an impaired uteroplacental circulation. Exclusion criteria: women with DM, systemic LE, or other connective tissue diseases; women with whom it had already been agreed previously that they would be treated with low-dose aspirin because of their obstetric history. PIH was defined as an increase in diastolic blood pressure of at least 25 mmHg in the course of pregnancy, with a final DBP.	3 g 12-14 weeks until delivery	No ≠ between the intervention group and the placebo group.

Onwude et al. (1995) [77]	Intervention group: EPA and DHA Control group: placebo	Inclusion criteria: multigravida with (i) a history of one or more small babies, defined as birthweight less than the 3^rd^ centile; (ii) a history of proteinuric or nonproteinuric PIH, defined as hypertension (with or without proteinuria) developing during pregnancy, labour, and puerperium in a previously normotensive nonproteinuric woman; and (iii) a history of unexplained stillbirth.	1.62 g 1.08 g 16-20 weeks until 38 weeks of gestation	No ≠ between groups.

Salving et al. (1996) [137]	Intervention group: fish oil Group control: control of the consumption of olive oil	Inclusion criteria: all women attending the main midwife clinic in the city of Aarhus, Denmark, prior to 30 weeks of gestation. Exclusion criteria: women with a history of placental abruption in an earlier pregnancy or a serious bleeding episode in the present pregnancy. Women using prostaglandin inhibitors regularly and allergy to fish and regular intake of fish oil.	2.7 g *ω*-3 30 weeks of gestation until delivery	No ≠ on systolic or diastolic blood pressure.

Smuts et al. (2003) [138]	Intervention group: eggs enriched with DHA Control group: common egg	Inclusion criteria: pregnant women 16–36 years of age; 24–28 weeks of gestation, at enrollment. Able and willing to consume eggs. Access to refrigeration. Plan to deliver at Truman Medical Center Singleton gestation. Exclusion criteria: <16 or >36 years of age. Weight > 240 lb at baseline. Serious illness such as cancer, LE, and hepatitis, known to have any untreated infectious disease, DM, or GDM at baseline. Elevated blood pressure attributed to any cause.	133 mg/unit 33 mg/unit 24-28 weeks of gestation until delivery (12 eggs/week of study)	↑: duration of gestation in the intervention group

Olsen et al. (2000) [139]	Intervention group: fish oil Group control: olive oil	Inclusion criteria: women after 16 weeks of gestation with an uncomplicated pregnancy, who in an earlier pregnancy had experienced (a) preterm delivery (before 259 days of gestation), (b) IGR (<5th centile), (c) PIH (DBP > 100 mmHg), and (d) women with current twin pregnancies (trial D). Exclusion criteria: DM in or before pregnancy; diagnosed severe fetal malformation or hydrops in current pregnancy; suspicion in current pregnancy, or occurrence in an earlier pregnancy, of placental abruption; drug or alcohol abuse; regular intake of fish oil or of nonsteroidal anti-inflammatory agents or other drugs with an effect on thrombocyte function or eicosanoid metabolism; and allergy to fish products.	2.7 g *ω*-3 (prophylactic) or 6.1 g *ω*-3 (therapeutic) 20-33 weeks until delivery	↓: risk of recurrence of preterm labor in the intervention group *vs.* control.

Legend: CD: cardiovascular disease; DBP: diastolic blood pressure; DHA: docosahexaenoic acid; DM: diabetes mellitus; EPA: eicosapentaenoic acid; GDM: gestational diabetes mellitus; GLA: gamma-linolenic acid; IUGR: intrauterine growth restriction; LE: lupus erythematosus; MAP: mean arterial pressure; PGE2: prostaglandin type E2; PIH: pregnancy-induced hypertension; RD: renal disease; SAH: systemic arterial hypertension; *ω*-3: omega-3; ↑: higher; ↓: lower; ≠: difference.
